# Behavior change support in the new era of obesity care

**DOI:** 10.1186/s12916-026-05167-2

**Published:** 2026-08-28

**Authors:** Brunna Boaventura, Stuart W Flint

**Affiliations:** 1 Department of Nutrition, Health Sciences Center, Federal University of Santa Catarina, Florianópolis, Santa Catarina Brazil; 2 School of Psychology, University of Leeds, Leeds, UK

**Keywords:** Comprehensive obesity care, Interdisciplinary approach, Weight stigma, Behavior change, Psychosocial risks

## Background

Evidence of the effectiveness of glucagon-like peptide-1 receptor agonists (GLP-1 RAs) to improve cardiometabolic health has increased rapidly, as has patient and public demand. This has, and continues to, reshape clinical practice and public health priorities. The World Health Organization recognizes obesity as a chronic and relapsing disease that requires a multimodal, continuous, and person-centered care model, based on behavioral, pharmacological, and, when indicated, surgical interventions [1]. Lifestyle and behavioral interventions are an ever-present component in obesity management, however, their implementation in routine care remains inadequate, often reduced to brief advice or general education rather than structured, sustained support [2]. This implementation gap reflects limited clinician training, weak referral pathways to allied healthcare professionals providing counseling, coaching and ongoing support, inadequate reimbursement, and care models that fail to address the psychological, social, and structural barriers to behavior change [2]. These limitations become even more important as GLP-1 RAs become increasingly available through clinical, public, private, and unregulated pathways, exposing gaps in access, regulation, and clinical supervision, including self-directed use, unregulated online sales, and counterfeit or falsified products [3].

This Comment does not aim to revisit the established role of lifestyle interventions in obesity treatment, but to examine the need to strengthen behavior change support in the context of the current expansion of GLP-1 RA use, considering the complexity of behavior change in obesity management, the consequences of weight stigma, and the psychosocial risks of self-directed or unregulated access that may limit comprehensive, person-centered care.

### The complexity of behavior change in obesity management

Although GLP-1 RAs are associated with health benefits beyond weight management, sustaining long-term outcomes requires behavioral and psychosocial support. This is particularly pertinent given the variability in treatment responses. In this context, medications delivered without multimodal and multidisciplinary support may represent incomplete and unsafe obesity care [1, 3]. Previous research has reported that discontinuation of medications for obesity was followed by weight regain and reversal of cardiometabolic benefits [4]. In a systematic review and meta-analysis of 37 studies including 9,341 participants, West et al. reported that weight regain after medication cessation was faster than weight regain after behavioral weight management programs, independent of initial weight loss [4].

Behavior change support refers to sustained, person-centered interventions integrated within chronic care programs to facilitate the initiation and maintenance of health-related behaviors through counseling, goal setting, and ongoing monitoring and support [1]. Health behaviors are influenced by the interaction of multiple psychological determinants operating within social and structural contexts, including cognitive, affective, motivational, and self-regulatory processes that govern whether intentions are translated into action [5]. Overlooking this complexity remains a key culprit in the persistence of reductionist and suboptimal treatment models, with weight stigma and discrimination perpetuating a psychosocial burden and disengagement from care in people with obesity.

### Weight stigma consequences and the need for comprehensive obesity care

Although efforts have focused on reducing weight stigma in healthcare, the conversation should now extend to addressing its consequences as a core component of behavior change support.

Weight stigma represents a major barrier in obesity care. Addressing weight stigma in obesity care has been associated with improvements in psychological and behavioral outcomes, including eating self-efficacy, quality of life, treatment acceptability, and reduction of internalized weight stigma [6]. These findings suggest that behavior change support should not only facilitate health-related behaviors but also improve coping mechanisms for the psychological and social consequences of obesity and weight stigma, including challenges that may persist beyond weight management.

It has been postulated that GLP-1 RAs may attenuate weight stigma by highlighting the biological drivers of obesity, such as satiety, appetite, and food reward, while facilitating behavior change [7]. However, people with obesity are frequently subjected to moral judgment for using GLP-1 RAs, reinforcing stigma in a cultural context where body weight is subject to moral appraisal rather than clinical understanding [8].

Meanwhile, intensive behavioral therapy programs, including those implemented by the Centers for Medicare & Medicaid Services, focus on diet, physical activity, and self-monitoring, with success defined by weight-loss thresholds and service delivery largely restricted to physicians and nurses [9]. This weight-centered focus precludes the integration of broader determinants of behavior change and is misaligned with current treatment goals, including cardiometabolic outcomes, quality of life, and quality-adjusted life years.

Obesity care must move beyond a prescriptive, educational model toward behavior change approaches that address the psychosocial aspects of obesity, including fears of weight regain, existential distress, moral injury, and identity-related challenges. Notably, weight loss does not necessarily resolve the psychological and social consequences of experiencing and internalizing weight stigma, including body image concerns, reduced self-concept, and adaptation to bodily changes [10]. These may affect self-efficacy, quality of life, engagement with care, and the ability to sustain health-related behaviors. This concern is intensified in the context of increasing demand for obesity medications, as cost and access barriers may lead some patients toward self-directed, unregulated, and weight-centered treatment pathways, where fragmented care can leave these psychosocial needs insufficiently addressed [3].

## Conclusions

As obesity medications become increasingly embedded in public and private health systems and accessible through self-directed, unregulated pathways, cost and access barriers may lead patients to prioritize medications access over comprehensive care. Simply prioritizing medications access may reinforce a weight-centered approach and leave the behavioral, psychosocial, and multidisciplinary support needed for safe and sustained outcomes insufficiently addressed.

Although multidisciplinary and multimodal care is recommended for obesity management, its implementation remains limited. This underscores the need for investment in public health programs capable of delivering integrated care. Even in the private sector, access to such care is often limited, as the combined cost of multiple health professionals and pharmacological treatments makes this approach unaffordable for most.

Policy responses, health systems, and regulators must ensure that all obesity services, including those in the private sector, provide access to appropriately trained multidisciplinary healthcare professionals, recognizing that pharmacotherapy alone cannot deliver sustainable outcomes without integrated behavioral, psychological, and social support, which should also be available to empower patients to enable and maintain their health.

## Data Availability

No datasets were generated or analysed during the current study.
